# Addition of Bone Marrow Aspirate Concentrate Resulted in High Rate of Healing and Good Functional Outcomes in the Treatment of Clavicle Fracture Nonunion: A Retrospective Case Series

**DOI:** 10.3390/jcm10204749

**Published:** 2021-10-16

**Authors:** Dvir Benshabat, Shai Factor, Eran Maman, Amal Khoury, Raphael Krespi, Itay Ashkenazi, Ofir Chechik, Oleg Dolkart

**Affiliations:** Division of Orthopedic Surgery, Tel Aviv Sourasky Medical Center, Tel Aviv University Sackler Faculty of Medicine, Weizmann St 6, Tel Aviv 6423906, Israel; dvirbs@gmail.com (D.B.); eemaman@gmail.com (E.M.); amalkh@tlvmc.gov.il (A.K.); rkrespi@gmail.com (R.K.); itay.ashkenazi@gmail.com (I.A.); ofirchik@yahoo.com (O.C.); dolkarto@gmail.com (O.D.)

**Keywords:** bone marrow aspirate concentrate, BMAC, clavicle fracture, nonunion, open reduction, internal fixation

## Abstract

Bone marrow aspirate concentrate (BMAC) is an autologous cell composition that is obtained through a needle aspiration from the iliac crest. The purpose of this study was to evaluate the outcomes of patients treated with open reduction and internal fixation with BMAC supplementation for clavicle fracture nonunion. This was a retrospective case series of 21 consecutive patients with clavicle fracture nonunion that were treated with ORIF and BMAC supplementation between 2013 and 2020. Patients were evaluated for fracture union, time to union, complications related to surgical and donor site, and functional outcome using the Quick Disability of the Arm Shoulder and Hand (QDASH), subjective shoulder value (SSV), and pain. The mean age was 41.8 years. The mean follow-up was 36 months. Twenty (95.2%) patients demonstrated fracture union, with a mean time to union of 4.5 months. Good functional scores were achieved: SSV, 74.3; QDASH, 23.3; pain level, 3.1. There were no complications or pain related to the iliac crest donor site. Supplementary BMAC to ORIF in the treatment of clavicle fracture nonunion is a safe method, resulting in high rates of fracture union and good functional outcomes with minimal complications and pain.

## 1. Introduction

Clavicle fractures comprise 2.6–5.0% of all fractures in adults, with an annual incidence of 36.5 per 100,000. The vast majority (70–80%) occur in the middle-third [1,2,3,4,5]. Historically, most closed middle-third and distal clavicle fractures in adults have been treated conservatively; however, with conservative treatment, the risk for nonunion can reach as high as 44% for distal-third fracture, while the risk for symptomatic nonunion can reach as high as 24% for midshaft fractures [6,7,8,9,10,11].

Trends in the last decade have changed, and surgical treatment has become more prevalent, especially in young and active patients, resulting in lower rates of symptomatic nonunion in midshaft fractures (2.6%) [12]. Commonly accepted indications for surgical treatment of midshaft fractures are a displacement of over 100%, shortening > 2 cm at the fracture site, “Z-type” deformity, or comminution. For distal clavicle fractures, the commonly accepted indication for surgical treatment is a displaced type 2 (Neer classification) fracture with surgical treatment showing higher rates of radiographic union; however, outcomes of this treatment have not shown superiority over nonsurgical treatment. In both midshaft and distal fractures, no surgical technique has been shown to be superior [6,12].

Fracture nonunion is defined as a failure of healing in the expected time and very low likelihood of healing without further intervention. Delayed union is defined as a fracture that has not completely healed in the time expected, but still has the potential to heal without further intervention. Establishment of a nonunion is arbitrarily defined at 6–8 months. Nonunion causes are numerous and may be divided according to host factors (e.g., age, endocrine disorders, exposure to radiation, and medications), injury pattern (e.g., high energy and open fracture increase risk for nonunion), and mechanical stability. Nonunion is classified according to the relative biologic activity at the fracture site and the presence of infection (septic nonunion). Atrophic and oligotrophic nonunion represent poor biologic potential for healing. Hypertrophic nonunion is an adequate healing response with lack of adequate stability. Classification is usually done using radiographic analysis. When nonunion occurs in clavicle fractures, the standard treatment is direct plate fixation, with or without bone grafting [13,14,15,16,17,18,19].

Bone marrow aspirate concentrate (BMAC) is an autologous cell composition containing nucleated cells found in bone marrow, which is obtained through a needle aspiration from the iliac crest. Autologous bone graft provides both osteo-inductive and osteoconductive qualities. Thus, ICBG is considered the gold standard in the treatment of fracture nonunions. However, in the setting of clavicle fracture nonunion, when bone loss and/or shortening are minimal and structural bone graft is often not required, BMAC can provide osteo-inductive qualities and requires less time to obtain, with lower morbidity and pain in the donor site [20].

In a systematic review of the clinical applications of BMAC done by Gianakos et al. [21], they reviewed eight studies on the use of BMAC in nonunion and delayed union and found that it shortens time to union and decreases complications with a comparable to better union rate when compared to the use of ICBG. None of the studies reviewed examined the effect on clavicle fractures nonunion [21].

This study focuses on the proposition that treating clavicle fracture nonunion with open reduction and internal fixation with supplementation of BMAC will result in high rates of union, satisfactory functional and pain scores, and minimal donor site pain and/or complications. Consequently, after approval by the institutional ethics and a scientific committee, we conducted a retrospective study to evaluate the outcome of patients treated with this technique at our institution.

## 2. Methods

From 2013 to 2020, 22 consecutive patients with middle and distal third clavicle fracture nonunion were treated with ORIF and BMAC supplementation in our institute. Operation was indicated in these cases due to symptomatic nonunion, with pain and/or pathologic movement at the fracture site being the main symptoms. For all patients, the diagnosis of nonunion was confirmed during the surgical intervention. Nonunion was defined as either failure of radiographic union after operative treatment or after a minimum of 3 months following conservative treatment. After approval by the institutional ethics and scientific committee, patient data were collected from medical charts, a telephonic interview was performed, and the cohort was then divided into two groups according to the treatment they received for their initial injury (surgical vs. conservative). Only closed middle-third and distal-third fracture nonunions that were radiographically and intraoperatively evident were included. Open fracture, concomitant neurovascular injuries, or infected nonunion were excluded. No history of immunological disease or use of steroids was noted. No history of contralateral clavicle fracture was noted in any of the cases.

### 2.1. Surgical Technique

**Figure 1:**
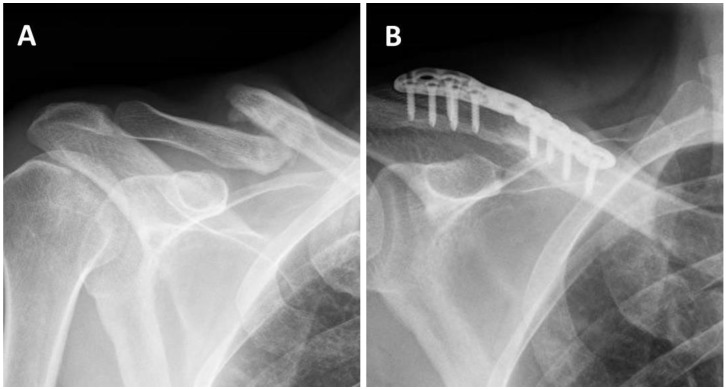
A 47 year old patient who suffered from symptomatic right–middle clavicle nonunion after conservative treatment: preoperative X-ray (**A**) at 5 months after the injury, and (**B**) at 1 year following ORIF with supplementation of BMAC. ORIF; open reduction and internal fixation BMAC; bone marrow aspirate concentrate.

All operations were performed by a fellowship-trained shoulder specialist, under general anesthesia, set in the standard beach-chair position; antibiotic prophylaxis was given in the form of 2000 mg cefamizine that was administered IV preoperatively and continued postoperatively for 24 h. For the BMAC acquisition, a small area over the iliac crest donor site was draped; a unique five-port aspiration needle with trocar from the BMAC2 Concentration kit was inserted percutaneously into the iliac crest, and 60 mL of bone marrow blood was aspirated and processed with the SmartPrep 3 system (Terumo Harvest, Tokyo, Japan) according to the manufacturer’s instructions, obtaining roughly 10 mL of bone marrow concentrate. The skin incision was made upon the previous incision in Group Surg and extended if needed. The nonunion site was exposed, while interposed fibrous tissue and callus were debrided to achieve bleeding healthy bone. The medullary canal was then opened and reamed with a 3 mm drill. Decortication of the proximal and distal fragments was performed using a periosteal elevator on the superior surface to prepare for plate application; the surrounding periosteal sheaths were protected in order to preserve the circulation. Fixation was done with the designated Stryker VariAx clavicle plating system with 2.7 mm or 3.5 mm locking/nonlocking screws (Stryker, Kalamazoo, MI, USA) or with the Acumed Clavicle Plating system with 2.3 mm or 3.5 mm locking/nonlocking screws (Acumed, Hillsboro, OR, USA) (Figure 1). Long enough plates were used to ensure three screws in both sides of the fracture. The wound was closed in layers, with an attempt to close the periosteum over the plate, when possible, followed by closure of the platysma and fascia. At this point, the marrow concentrate was injected into the fracture site, in hopes of creating a closed compartment in which the marrow concentrate would engulf and penetrate the fracture site. Subcutaneous and skin closures were done in a standard fashion with dissolvable sutures.

Postoperatively, the patient’s arm was held in a sling. Passive and active mobilization up to 90° forward flexion and abduction for the first 2 weeks, full range of motion after first checkup at 2 weeks, and strengthening after second follow-up at 8 weeks post-op were allowed and encouraged as tolerated. Gradual return to sports and manual labor were allowed after clinical and radiographic union.

### 2.2. Clinical Outcome

Postoperatively, patients were examined at 2, 8, 20, and 50 weeks and then annually as needed, and radiographic evaluation with standardized anteroposterior and clavicle view was performed on every follow-up visit. Union was defined as a completely bridging bone in two radiographs (i.e., two healed cortices), with obliteration of the fracture gap; this method was described in previous studies [22,23,24]. All radiographs were evaluated independently by two senior authors (D.B. and O.C.).

Functional outcomes were evaluated using the subjective shoulder value SSV and the Quick Disabilities of the Arm, Shoulder, and Hand (QDASH). Pain level was measured using the visual analogue scale (VAS) for both shoulder and iliac crest donor site; questions were asked regarding complications at the iliac crest donor site.

### 2.3. Statistical Analysis

All patients were measured for all response variables, which included demographic variables and outcomes. Data were presented as means and standard deviations for continuous response variables or percentages for discrete variables. Descriptive statistics only were used to describe the basic features of the data in the current study. Statistical analysis was carried out by the SPSS for Windows software, version 22.0 (Chicago, IL, USA)

## 3. Results

One patient, with a history of drug abuse, was lost to follow-up 2 weeks post-op and was excluded from the cohort; the remaining 21 patients composed the study population. The mean age was 41.8 years (range 26–68); there were 15 males and six females; nine were smokers, and three were NEER type 2 distal clavicle fractures.

Fourteen patients were diagnosed with clavicle fracture nonunion following conservative treatment for their initial injury (group Con), and seven were diagnosed following surgical treatment for their initial injury (group Surg). The mean age in group Con was 45 years (range 26–68); there were 10 males and four females; six were smokers, and two were NEER type 2 distal-third clavicle fractures. The mean duration of conservative treatment was 6.9 months (range 3–15). Group Con was considerably homogeneous in terms of initial management.

Group Surg consisted of seven patients who were treated with ORIF initially for their acute injury, and the course of their management was considerably more heterogenic compared to group Con. The mean patient age was 35 years (range 30–45); there were five males and two, females and three were smokers. Two patients had suffered polytrauma, two were refractures, one was a distal clavicle NEER type 2 fracture, one was treated with a structural fibular allograft due to substantial bone deficit and shortening, and one patient was treated with BMAC after two previously failed ORIF operations without a bone graft.

The mean follow-up period was 36 months (range 8–82) for the entire cohort, 34.43 months (range 8–67) in group Con, and 39.43 months (range 16–82) in group Surg.

Twenty (95.2%) patients demonstrated fracture union, with a mean time to radiographic union of 4.5 months (range 2–14). The patient that did not achieve union at final follow-up at 20 months was a smoker, suffered a NEER type 2 distal clavicle fracture, and was initially treated conservatively (group Con). He was offered a revision surgery but declined. All the remaining patients from both groups achieved union uneventfully.

Patient demographics are presented in Table 1.

Union rate was 100% for all middle-third fractures. The mean time to union for the entire cohort was 4.5 months (range 2–14), while it was 3.9 months (range 2–10) in group Con and 5.7 months (range 3–14) in group Surg (Table 2). One patient had her plate removed after union of the fracture due to hardware prominence. No refracture developed. All patients reported no pain or complications at the donor site. The mean shoulder pain level (VAS) was 3.1 (range 0–9) for the whole cohort, 3.7 (range 0–9) in group Con, and 2.0 (range 0–6) in group Surg. The mean SSV scores were 74.3 (range 10–100) for the entire cohort, 71.9 (range 10–100) in group Con, and 78.6 (range 60–100) in group Surg. The mean QDASH scores were 23.3 (range 0–63.6) for the entire cohort, 25.2 (range 0–63.6) in group Con, and 19.9 (range 2.3–54.6) in group Surg (Table 3).

## 4. Discussion

The aim of this study was to evaluate the outcome of ORIF supplemented with BMAC for the treatment of clavicle fracture nonunion. A series of 21 patients were included in this study with the main results being a 95.2% union rate with no complications at the donor site.

Clavicle fracture nonunion is an infrequent complication to a frequent injury. Nonoperative treatment of all-site fracture nonunion, such as low-intensity pulsed ultrasound (LIPUS) and extracorporeal shock wave therapy (ESWT), shows reasonable outcomes and fracture healing. In their review from 2017, Leighton et al. [25] estimated union rates using LIPUS at 82% (77–87%), while Romano et al. [26] estimated them to be 70–93%. Moya et al. [27] reviewed in 2018 the role of ESWT in treating fracture nonunions and showed union rates of 63–87%. The main advantage of these technologies is the ability to avoid the need for additional complex operations for the treatment of nonunion. LIPUS and ESWT may be most useful for patients at high risk for surgery. However, to our knowledge, there are no studies assessing the efficacy of LIPUS or ESWT in the treatment of clavicle fracture nonunion. There are some case reports including patients with clavicle fracture nonunion, but they are scarce, and the number of patients is relatively small. Nolte et al. [28] reported 29 cases of nonunion treated with LIPUS with only one patient with clavicle fracture nonunion. With not enough evidence to support nonoperative treatments to date, management of clavicle fracture nonunion remains operative [29].

Previous studies regarding surgical treatment for clavicle fracture nonunion are retrospective in nature and describe small cohorts similar in size to this study. One of the largest studies was published in 2015 by Schnetzke et al., [30] in which they retrospectively compared treatment of clavicle fracture nonunion treated by ORIF with or without BG in 58 patients. Their long-term follow-up of 8.9 years showed an advantage in the BG group, with 93% union rates compared to only 73% in the non-BG group. In 2017, Rollo et al. [31] published their series of 57 patients achieving a union rate of 98% and concluded there was a necessity of autogenous or allogeneic bone grafting for patients with an atrophic nonunion. In 2014, Faraud et al. [22]. achieved a 90.5% union rate in a series of 21 cases and concluded that union is achieved with stable fixation with bone grafting from the site itself or the iliac crest. Other studies with smaller cohorts showed similar results with union rates of 94–100% with the use of BG [32,33,34,35]. In 2011, Singh et al. [36] compared the use of ICBG in 20 patients to demineralized bone matrix (DBM) in 10 patients as supplementation to ORIF in the treatment of mid-shaft clavicle nonunions; both groups achieved 90% union, and they concluded that DBM obviates the need for BG. In 2012, Huang et al. published two retrospective studies [23,24]. The first study described the treatment of 51 patients with hypertrophic nonunion treated with ORIF and BG from local callus, while the second study compared treatment of 60 patients with atrophic nonunion with ORIF alone vs. ORIF with ICBG. In both studies, all patients achieved union. Huang et al. [23,24] concluded that the use of autologous ICBG was not required to achieve union. The union rate achieved (95.2%) in the current study is well within the range of union rates found in previous studies of 90–100%.

Bone grafting is a commonly performed surgical procedure to augment bone regeneration in the treatment of nonunion; autologous bone graft (BG) remains the ‘gold standard’, and the iliac crest is most common harvesting site [37]. However, harvesting of iliac crest bone graft (ICBG) is associated with morbidity and several complications. In a large systemic review of 157 studies by Dimitriou et al. [38], the rate of complications after ICBG harvesting was found to be 19.37% (1249 complications in 6449 patients); the most common complications found were pain and sensory disturbances at the donor site some lasting over 2 years after surgery. The documentation and classification of these complications differed in the studies reviewed (major/minor, acute/chronic) with major complications defined as those that require further treatment, chronic donor site pain for 6–12 months, or prolonging the hospital stay (e.g., deep infection and iliac fracture). The difference in the overall morbidity rates between anterior and posterior harvesting sites was not statistically significant. Complications at the donor site after BMAC are rarely reported; Garnavos et al. [39] reported mild donor site discomfort in all five patients in the first 2 days after surgery. Gianakos et al. [21] found lower or no complications at the donor site using BMAC compared to other BG methods. Hernigou et al. [40] compared the use of BMAC to ICBG in two matched groups of patients with diabetes for ankle nonunion; they reported 11% complications in the ICBG group including major complications (e.g., deep infection, large hematoma, and persistent pain) compared to only 2% (mild complications including temporary sensory loss and mild pain) in the BMAC group. Of the clavicle fracture nonunion studies mentioned before, two reported ICBG donor site complications; Rollo et al. reported two (5%) patients that complained of chronic (>12 months) pain at the donor site [31], while Ebraheim et al. [35] reported one (7%) patient that complained of hematoma and chronic pain at the donor site. In this study, there were no donor site complications, and pain scores were 0 on the VAS scale with the BMAC technique.

Time to union in this study was 4.5 months for the whole cohort and 3.9 months for the group that was initially treated conservatively. Of the clavicle fracture nonunion studies mentioned before, five reported time to union. Schnetzke et al. reported a mean time to union of 4.3 months in the group treated with ORIF and BG and 10.3 months in the group treated with ORIF alone [30]. Rollo et al. reported a mean time to union of 14 weeks [31]. Nikiforidis et al. reported a mean time to union of 3–7 months in their series [32], Khan et al. reported a mean time to union of 5.3 months in their series [34], and Wenz et al. reported a mean time to union of 14 weeks in his young (aged 18–33 years) population of athletes [33]. Time to union in this study falls within the spectrum reported in earlier studies in which BG was used. It is the authors’ opinion that time to union is greatly influenced by the postoperative follow-up regimen of clinical and radiographic checkups, occurring at 2, 8, 20, and 50 weeks postoperatively. Most of this study’s patients achieved radiographic union at their third visit (20 weeks). It is likely that some patients achieved fractures union between 8 and 20 weeks, but radiographs were not obtained to prove it.

On the functional and pain aspect of the treatment, the final QDASH, SSV, and VAS scores were recorded and presented in this study. Of the clavicle fracture nonunion studies mentioned before, four reported their functional outcomes using the DASH/QDASH score. Schnetzke et al. reported a mean DASH score of 11.7 in the ORIF and BG group vs. 21.4 in the group treated with ORIF alone at a mean follow-up of 8.9 years [30], Rollo et al. reported a mean DASH score of 16.7 at a mean follow-up of 42 months [31], Faraud et al. reported a mean QDASH score of 17 at a mean follow-up of 41 months [22], and Khan et al. reported a mean DASH score of 24 at a mean follow-up of 34 months [34]. The mean QDASH score of 23.3 at a mean follow-up of 34 months reported in this study falls within the spectrum reported in earlier studies. SSV and VAS scores in the clavicle fracture nonunion literature are scarce and, therefore, could not be compared to this study’s results.

Addition of BMAC to ORIF in treating clavicle nonunion may have a negative effect by resulting in clavicle shortening due to the excision of structural bone graft during surgery to achieve bleeding healthy bone. However, several previous studies have found that clavicle shortening does not affect limb function, even when shortening exceeds 2 cm [41]. Moreover, previous studies have shown good clinical outcomes when treating clavicle nonunion with supplemental BMAC to ORIF [23,36]. Both treatment regimens have the same possible effect of clavicle shortening due to a lack of structural bone graft, which did not have clinical significance.

## 5. Limitations

Limitations of this study include its retrospective nature, the small sample size, and the grave heterogeneity of group Surg. Although it was initially contemplated by the authors, due to this heterogenicity, it was decided that a statistical comparison of the groups would not be done since it would have not yielded any clinically significant evidence.

## 6. Conclusions

Supplementary BMAC to ORIF in the treatment of clavicle fracture nonunion is a safe method, resulting in high rates of fracture union and good functional outcomes with minimal complications and pain.

## Figures and Tables

**Table 1:** Patient demographics.

	Nonunion after Conservative Treatment (*n* = 14)	Nonunion after Surgical Treatment (*n* = 7)	Total (*n* = 21)
Age (years) (SD) ^1^	45 (14)	35 (5.4)	41.8 (12.7)
Gender, Male (%)	10 (71)	5 (71)	15 (71)
Smoking (%)	6 (43)	3 (43)	9 (43)
Distal clavicle fracture (%)	2 (14)	1 (14)	3 (14)
Follow-up (months) (SD) ^1^	34.4 (15.5)	39.4 (26.3)	36.1 (19.2)

^1^ Values are presented as means and standard deviations.

**Table 2:** Primary outcome, union.

	Nonunion after Conservative Treatment (*n* = 14)	Nonunion after Surgical Treatment (*n* = 7)	Total (*n* = 21)
Nonunion (%)	1 (7.1)	0 (0)	1 (4.8)
Time to union * (Months) (SD) ^1^	3.9 (2.33)	5.7 (3.82)	4.5 (2.96)

^1^ Values are presented as means and standard deviations. * Excluding patient 2′ who did not achieve union.

**Table 3:** Pain and functional questionnaires.

	Nonunion after Conservative Treatment * (*n* = 13)	Nonunion after Surgical Treatment (*n* = 7)	Total (*n* = 20)
Shoulder VAS (SD) ^1^	3.7 (3.66)	2 (2.52)	3.1 (3.34)
SSV (SD) ^1^	71.9 (29.41)	78.6 (16.76)	74.3 (25.41)
QDASH (SD) ^1^	25.2 (25.18)	19.9 (18.06)	23.3 (22.59)
Donor site VAS	0	0	0
Donor site complications	0	0	0

^1^ Values are presented as means and standard deviations. * Excluding patient 11′ who was unavailable to answer the questionnaires.
